# Neuroimmune Regulation by TRPM2 Channels

**DOI:** 10.3390/cells15010076

**Published:** 2026-01-01

**Authors:** Xuming Zhang, Mitali Malhotra

**Affiliations:** School of Life Sciences, University of Warwick, Coventry CV4 7AL, UK

**Keywords:** TRPM2, ion channels, neuroimmune interaction, chronic pain, epilepsy, stroke, neurodegenerative diseases, temperature sensing

## Abstract

Mutual interaction between the nervous and immune systems underpins many pathophysiological processes. Transient Receptor Potential Melastatin 2 (TRPM2) channels are abundantly expressed in both systems, acting as a critical interface of neuroimmune interaction. TRPM2 channels in immune cells participate in innate immunity and immune inflammation by acting as an oxidative stress and metabolic sensor. TRPM2 in neurons functions not only as an oxidative sensor but also a temperature sensor and a pain transducer critical to neuronal death, temperature sensing, thermoregulation, and chronic pain. Cooperation between immune and neuronal TRPM2 influences the outcome of neuroimmune interaction and many diseases such as infection, inflammation, ischemic stroke, pain, and neurodegenerative diseases. Improved understanding of neuronal and immune TRPM2 interaction is essential for therapeutic interventions for the treatment of diseases mediated by TRPM2 channels.

## 1. Introduction

The nervous and immune systems are common in that both systems detect harmful stimuli, invading pathogens and injured tissues in the body and environment. They deploy coordinated host defence responses to remove danger and restore tissue homeostasis once activated. The coordinated neuroimmune interaction relies on shared receptors present on both systems [1,2]. For example, Toll-like receptors (TLRs) are expressed in both the immune cells and peripheral nociceptors, allowing them to trigger cooperative antimicrobial responses [2].

**Figure 1:**
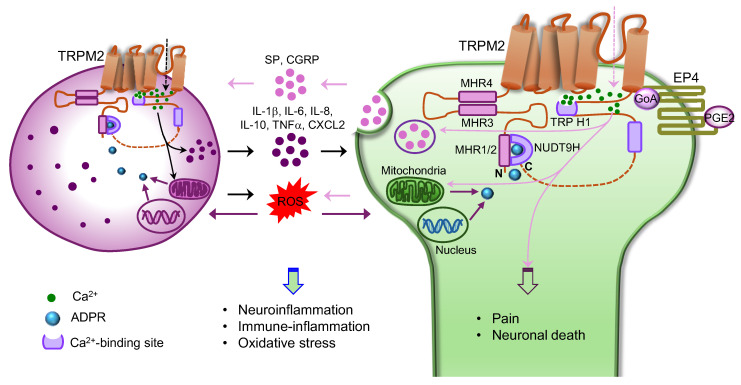
Neuroimmune interaction mediated by TRPM2 channels. The N-terminus of TRPM2 contains MHR1-4 domains and the C-terminus contains a Ca^2+^ binding site near the TRP H1 domain. The dotted line in the C-terminus of TRPM2 denotes the unresolved structural region between D1165 and G1235. Oxidative stress activates TRPM2 through increased production of ADPR (cyan sphere) from mitochondria and nucleus in both immune (**left**) and neuronal cells (**right**). Activated TRPM2 causes Ca^2+^ influx, which then promotes production and release of cytokines and chemokines (IL-1β, IL-6, IL-8, IL-10, TNFα (tumour necrosis factor α) and CXCL2 (C-X-C motif chemokine ligand 2), purple circles) in immune cells, triggering immune inflammation. These released inflammatory mediators act on neurons causing neuronal sensitization. Sensitized neuronal cells, together with increased intracellular Ca^2+^ due to TRPM2 opening, release neuropeptides (pink circles) such as substance P (SP) and calcitonin gene-related peptide (CGRP). These neuropeptides then act on immune cells, further potentiating immune cell activation and driving neurogenic inflammation and immune-inflammatory responses, forming positive feedback. Furthermore, increased intracellular Ca^2+^ in immune and neuronal cells caused by TRPM2 activation induces reactive oxygen species (ROS) production in mitochondria, which then further promotes TRPM2 activation forming second positive feedback, leading to aggravated immune inflammation, neuroinflammation, oxidative stress, pain, and neuronal death. Furthermore, TRPM2 can also be opened by the activated Gαi/o protein subunit GoA (pink sphere) coupled to the prostaglandin E2 (PGE2) receptor EP4, enhancing neuronal activity and promoting chronic pain.

TRPM2 channels were also dominantly expressed in both the immune and nervous systems, though TRPM2 is broadly expressed throughout the body [3,4]. Different types of cells in the neuroimmune system express TRPM2 such as macrophages, neutrophils, lymphocytes, microglia, satellite glia cells, dorsal root ganglia (DRG), striatal neurons, and hippocampal neurons [3,4,5,6,7,8,9]. In fact, TRPM2 was initially identified in the human fetal brain cDNA library, called transient receptor potential canonical 7 (TRPC7) and long transient receptor potential channel 2 (LTRPC2), due to its structural similarity to TRP channels [4,10]. Like other TRP channels, TRPM2 is a non-selective cation channel permeable to Ca^2+^ and Na^+^. Different from other TRP channels, TRPM2 lacks voltage sensitivity, exhibiting a linear I–V relationship [4,9,11,12]. Notably, TRPM2 contains a unique characteristic NUDT9H domain in the C-terminus (Figure 1). This domain exhibits similarity to NUDT9 enzyme responsible for hydrolysing ADP ribose (ADPR), a metabolic product of nicotinamide adenine dinucleotide (NAD). The NUDT9H domain was therefore suggested to bind ADPR responsible for TRPM2 activation caused by ADPR shortly after cloning of TRPM2 [4]. Subsequent structural studies confirmed the idea but further revealed that ADPR also binds to the cleft formed by the TRPM homologue region 1/2 (MHR1/2) domain in the N-terminus [13]. Therefore, both the NUDT9H and MHR1/2 domains participate in ADPR binding and TRPM2 channel gating through forming extensive interactions via intra- and inter-subunit contacts (Figure 1) [13,14,15]. Activation of TRPM2 by ADPR forms the basis of TRPM2 as a metabolic and oxidative stress sensor, contributing to various neurological diseases and beyond such as cancer, atherosclerosis, hypertension, and ischemia/reperfusion (I/R) injury (see below) [16,17].

## 2. TRPM2 Activation

### 2.1. NAD-Derived Metabolites

NAD is an essential cofactor in oxidative phosphorylation and redox reactions in cells. It is also an important substrate for generating second messengers and posttranslational modifications such as ADP-ribosylation [18]. ADPR is one of the key downstream metabolites of NAD and was identified as the first agonist for TRPM2 channels [4]. Soon after, TRPM2 was also found to be activated by oxidative stress [9,19,20,21,22], acting like an oxidative stress (redox) and metabolic sensor. Oxidative stress may directly activate TRPM2 through oxidization of TRPM2 protein. But this possibility was not supported by several lines of evidence. First, hydrogen peroxide (H_2_O_2_) failed to activate TRPM2 in inside-out patches lacking cytosolic factors [11,23,24]. Secondly, H_2_O_2_-induced TRPM2 activation was not affected by the reducing agents that prevent direct oxidative modification of proteins [11]. Thirdly, point mutations in the NUDT9H domain of TRPM2 unresponsive to ADPR also lost the response to H_2_O_2_ [22]. It was then found that oxidative stress activated TRPM2 indirectly through increased production of ADPR catalyzed by the cooperation of poly(ADPR) polymerase (PARP-1) and poly(ADPR) glycohydrolase (PARG) signalling in the nucleus or through NADase in the mitochondria. In support of this idea, oxidative stress-induced TRPM2 activation was prevented by deleting PARP-1 or by pharmacological blockade of PARP-1 or by expressing ADPRase to degrade cytosolic or mitochondria ADPR [20,22,25]. Similarly to oxidative stress, TNFα was also suggested to activate TRPM2 indirectly through generation of ADPR [26]. However, there was no significant change in ADPR production in Jurkat T cells exposed to H_2_O_2_ [27], suggesting ADPR-independent mechanisms underlying TRPM2 activation by oxidative stress.

Interestingly, 2′-deoxyadenosine 5′-diphosphoribose (2′-deoxy-ADPR) was found to be an even more efficacious endogenous TRPM2 agonist with 10 times more efficacy compared to ADPR [27]. Importantly, 2′-deoxy-ADPR production was increased by H_2_O_2_ through CD38, a type II ectoenzyme, but not through the PARP-1/PARG pathway [27], arguing that 2′-deoxy-ADPR is a more physiologically relevant endogenous TRPM2 agonist produced during oxidative stress. The relative role of ADPR and 2′-deoxy-ADPR in oxidative stress-induced TRPM2 activation in different cellular contexts remains to be determined.

Soon after the discovery of ADPR as a direct TRPM2 agonist, cADPR was also found to activate TRPM2 [12,21]. cADPR is mainly produced from NAD after catalysis by ADP-ribosyl cyclase such as CD38 [28]. It has been controversial whether cADPR directly opens TRPM2 channels independently of other NAD-related metabolites. Some reported direct activation of TRPM2 channels by purified cADPR in cell-free excised inside-out patches [21,29], whereas others found that cADPR cannot directly activate TRPM2 [23,24,30]. It remains to be resolved whether the difference is caused by the contamination of cADPR with ADPR. NAD^+^ was also found to activate TRPM2 [11], but the activation was attributed to contamination of NAD^+^ with ADPR [21]. Furthermore, nicotinic acid adenine dinucleotide phosphate (NAADP) is a partial TRPM2 agonist with low affinity [23]. It is therefore unlikely that NAADP is an important TRPM2 activator in physiological condition.

### 2.2. Ca^2+^ Activation of TRPM2

Ca^2+^ is essential for the activation of TRPM2 by chemical agonists. TRPM2 currents were markedly increased in the presence of Ca^2+^ [31], but cannot be activated by chemical agonists without Ca^2+^ [32]. It was initially thought that Ca^2+^ activates TRPM2 indirectly through the Ca^2+^ sensor calmodulin [33,34]. Subsequent structural analysis revealed that the second and third transmembrane segments of TRPM2, together with TRP H1 helix in the C-terminus, form a direct Ca^2+^-binding site near the membrane-cytosolic border of the channel (Figure 1). Ca^2+^ binding triggers a tilt at TRP H1, which is then transmitted through the MHR4 domain to the cytosolic ADPR-binding domain at the bottom layer formed by the NUDT9H and MHR1/2 domains (Figure 1), leading to a global rotation of the entire cytoplasmic domain and channel opening [14,35]. Notably, the Ca^2+^-gating site is very close (~3 nm) to the Ca^2+^ entrance pore domain (Figure 1). This physical proximity supports the idea that increased local cytosolic Ca^2+^ near the pore caused by the initial channel opening enhances Ca^2+^ gating forming positive feedback driving robust channel activation [24]. The Ca^2+^-binding site therefore functions like a TRPM2 gating amplifier. However, neither ADPR nor Ca^2+^ alone is sufficient to activate TRPM2 channels [24]. ADPR and Ca^2+^ thus coactivate TRPM2 through concerted actions [35].

### 2.3. Temperature Activation of TRPM2

TRPM2 is closely related to TRPM8, a cold-sensitive ion channel. However, TRPM2 was not directly activated by cold but by heat temperatures above 40 °C in transfected HEK293 cells [12,24,36]. Different from oxidative stress, heat seems to directly gate TRPM2 channels independently of endogenous chemical agonists, because heat-activated TRPM2 channels in inside-out membrane patches lacking cytosolic factors [12]. However, in subsequent similar study employing inside-out membrane patches containing TRPM2 channels, heat alone was found to be insufficient to activate TRPM2 without co-presence of the channel agonists such as Ca^2+^ and ADPR [24], suggesting allosteric coactivation of TRPM2 channels by heat and channel ligands. The findings also suggest that the temperature activation threshold of TRPM2 can be shifted to lower physiological temperature ranges by chemical ligands.

Consistent with temperature activation of TRPM2, TRPM2 was proposed as a warmth sensor in sensory DRG neurons [7]. Mice lacing TRPM2 were defective in sensing innocuous warm temperatures, but their noxious heat sensing was normal [7,37]. Correspondingly, warmth (34–42 °C)-sensitive DRG neurons were reduced in DRG neurons [7,37]. However, heat (>42 °C)-sensitive DRG neurons were also reduced in TRPM2 null mice. In fact, over half of TRPM2-expressing DRG neurons were activated by noxious heat (>42 °C) [7]. The higher temperature activation threshold for TRPM2 in isolated DRG neurons was suggested to be due to the loss of endogenous factors that regulate the temperature sensitivity of TRPM2. However, in vivo trigeminal ganglia (TG) and spinal cord imaging revealed that most of warmth-sensitive DRG and spinal cord neurons are mediated by TRPV1^+^ (transient receptor potential vanilloid 1) neurons without much involvement of TRPM2 [38,39]. Furthermore, there was no change in the responses of skin nerves to warm temperatures in ex vivo skin nerve recordings from TRPM2-deficient mice [40]. Difference was also not found in TRPV1-lacking mice [40]. It was then proposed that warmth perception was not directly caused by warm-excited C-fibres, but instead mainly driven by TRPM8^+^ cool-sensitive C-fibres inhibitable by warm temperatures [40]. Overall, TRPM2 in DRG neurons acts as a warmth/heat sensor but warmth perception in animals requires cooperation of TRPM2 with TRPM8 and TRPV1.

## 3. TRPM2 in Thermoregulation

Apart from expression in the peripheral DRG neurons, TRPM2 was also expressed in the preoptic area (POA) in the hypothalamus in the brain. Hypothalamus TRPM2 was found to be activated by ~38 °C responsible for the warmth sensitivity of POA neurons [41]. Animals incubated at 37 °C and 45 °C exhibited a significant increase in c-Fos expression in TRPM2^+^ neurons in the POA, further supporting that TRPM2 responds to warm temperatures in the POA [42]. Behavioural studies demonstrated that body temperature and fever response were increased by inhibiting TRPM2^+^ POA neurons or by deleting the TRPM2 gene [41,43]. Interestingly, TRPM2 in the POA was also sensitive to ultrasound. Activation of TRPM2 by ultrasound induced a torpor state in animals resulting in hypothermia and hypometabolic state [44]. Altogether, TRPM2 plays an important role in fever response and thermoregulation. Whether TRPM2 in POA neurons is involved in warmth perception apart from thermoregulation remains unknown.

TRPM2 was also found to be expressed in pro-opiomelanocortin (POMC) neurons in the arcuate nucleus (ARC) in the hypothalamus, a brain region driving satiety signals promoting energy expenditure [45]. However, activation of TRPM2 in the POMC neurons increased core body temperature and brown adipose tissue (BAT) thermogenesis [45], opposite to the effect of TRPM2 in the POA. Furthermore, TRPM2 was expressed in the brown and white adipose tissues of mice. Cold exposure significantly increased TRPM2 expression in the adipose tissues and enhanced thermogenesis in a TRPM2-dependent manner, because TRPM2-knockout (KO) mice exhibited lower thermogenesis and energy expenditure during cold exposure [46]. Therefore, TRPM2 in different tissues exerts different and even opposing effects on body temperature.

## 4. TRPM2 in Innate Immunity and Inflammation

**Figure 2:**
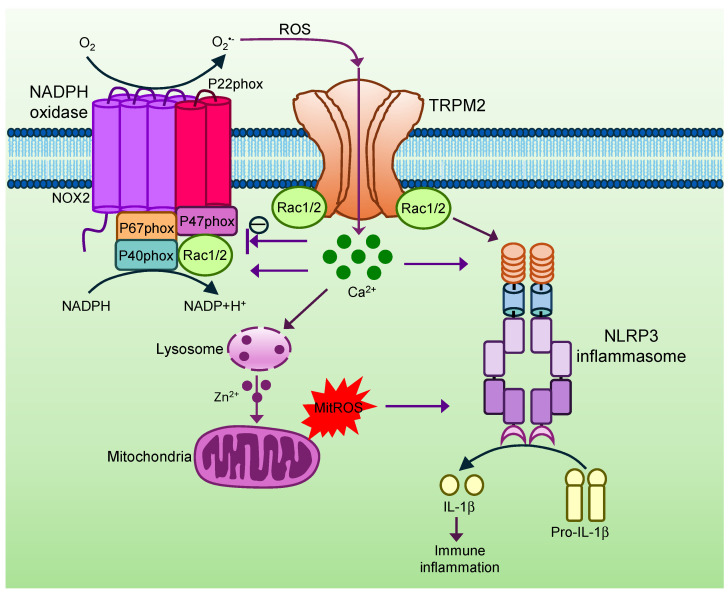
TRPM2 is a central regulator of NADPH oxidase and NLRP3 inflammasome. ROS generated from different sources such as NADPH oxidase, a large enzyme complex comprising NOX2, P22phox, p67phox, p47phox, and p40phox subunits, and Rac1/2 activate TRPM2 leading to Ca^2+^ flux and membrane depolarization. Depolarized membrane inhibits NADPH oxidase activity, forming negative feedback. However, increased Ca^2+^ and associated Rac1/2 with TRPM2 promote NADPH oxidase activity resulting in ROS generation, perpetuating TRPM2 activation. Increased intracellular Ca^2+^ also causes lysosome dysfunction and release of Zn^2+^, which further increases mitochondria ROS generation. Increased mitochondria ROS and Ca^2+^ along with associated Rac1/2 act together to activate NLRP3 inflammasome, leading to IL-1β release and immune inflammation.

Reactive oxygen species (ROS) are often generated during immune responses and tissue inflammation. ROS kill pathogens contributing to host defence while promoting the production of cytokines and chemokines, aggravating inflammatory responses. TRPM2 is a redox sensor abundantly expressed in the immune system such as monocytes, macrophages, lymphocytes, and microglia [9,11,47,48], suggesting that TRPM2 may mediate the effect of ROS. Indeed, TRPM2 was found to mediate the production of inflammatory cytokines and chemokines (IL-6, IL-8, IL-10, TNFα (tumour necrosis factor 2) and CXCL2 (C-X-C motif chemokine ligand 2)) in monocytes and macrophage caused by ROS and lipopolysaccharide (LPS) (Figure 1) [47,48]. Activation of TRPM2 by ROS also promoted activation of NLRP3 (nucleotide-binding and oligomerization domain (NOD)-like Receptor Protein 3) inflammasome and secretion of IL-1β in a Ca^2+^-dependent manner (Figure 2) [49], further aggravating inflammatory responses. Notably, TRPM2 interacted with Rac1, a small signalling G protein belonging to the Rho family of GTPase and was required for Rac1 activation induced by ROS [50]. Interestingly, recent research further demonstrated that activated Rac1/2 promoted NLRP3 inflammasome activation [51,52]. It is thus possible that TRPM2 promotes NLRP3 inflammasome activation also through Rac1/2 in addition to through Ca^2+^ (Figure 2). Consistent with these findings, the deletion of TRPM2 attenuated cytokines and chemokines (IL-1β, IL-6, TNFα and CXCL2) production and inflammation in acute and chronic colitis models in mice [47,53]. TRPM2 deletion also inhibited NLRP3 inflammasome activation and hepatic I/R injury [54]. In parallel, TRPM2 enhanced neutrophil infiltration by facilitating chemotaxis of neutrophils in response to chemokines and hydrogen peroxide through generating Ca^2+^ pulses in the leading edge of neutrophils [55,56,57]. Furthermore, Ca^2+^ signals generated by TRPM2 increased mitochondria ROS production through dysregulating lysosome and the release of Zn^2+^ (Figure 1 and Figure 2) [58]. The generated ROS could then in turn activate TRPM2, forming a feed-forward vicious positive cycle between ROS and TRPM2 (Figure 1) [16,59]. TRPM2 is therefore not only a ROS sensor but also a ROS generator and amplifier. Taken together, TRPM2 employs multiple mechanisms to promote tissue inflammation.

These findings indicate that TRPM2 is proinflammatory and harmful. The proinflammatory action of TRPM2 is also consistent with the subsequent finding that TRPM2 favours macrophage polarization towards the M1 phenotype [60,61]. However, others reported that TRPM2 is protective and beneficial through combating bacterial infection, exerting anti-inflammatory effect. They found that TRPM2 switched chemotactic neutrophil to microbial killing in response to ROS generated by neutrophils [62]. At the system level, TRPM2-lacking mice were more susceptible to bacterial infection leading to increased bacterial burden and higher mortality rate [60,61,63]. Unexpectedly, the deletion of TRPM2 even enhanced the production of ROS and cytokines (IL-1α, IL-6, IL-10 and TNFα) in phagocytes and macrophages and facilitated M1 macrophage polarization and infiltration of monocytes and neutrophils, aggravating tissue inflammation [62,64,65,66,67]. The anti-inflammatory effect of TRPM2 was thought to be caused by inhibiting NADPH (nicotinamide adenine dinucleotide phosphate) oxidase activity through depolarizing membrane potentials in phagocytes (Figure 2), leading to reduced ROS production [64,65,66]. Based on these findings, ROS activates TRPM2, which then inhibits NADPH oxidase and ROS generation, forming negative feedback (Figure 2).

Altogether, TRPM2 is a crucial bidirectional regulator of inflammation and innate immunity. The pro- and anti-inflammatory effects of TRPM2 may be due to different cellular and tissue contexts, inflammation models, and different inflammatory stages. Furthermore, immune inflammation may also be affected by neuronal TRPM2, depending on different extents of nerve innervation in targeted tissues. However, the role of neuronal TRPM2 was mostly disregarded in previous research, which may lead to different effects of TRPM2 on immune inflammation.

## 5. TRPM2 in Neurological Diseases

### 5.1. Chronic Pain

Chronic pain evolves as a result of mutual interaction between the immune and nervous systems [1,68]. TRPM2 was expressed in both the immune cells and sensory DRG neurons, playing a critical role in different types of chronic pain [69].

#### 5.1.1. Chronic Inflammatory and Neuropathic Pain

TRPM2 has been implicated in chronic inflammatory pain, osteoarthritis pain, and neuropathic pain [69,70]. Interestingly, TRPM2 expression level was correlated with damage and ROS levels in sciatic nerves in diabetic neuropathic pain [71], suggesting a role for TRPM2 in diabetic neuropathy.

Mechanical and heat hyperalgesia in carrageenan-induced inflammatory pain and nerve injury-induced neuropathic pain were reduced in TRPM2-KO mice. The effect was thought to be indirectly mediated by the proinflammatory effect of TRPM2 in inflammatory cells but not directly through TRPM2 channels on sensory neurons, because neutrophil and microglia accumulation and CXCL2 production were reduced in TRPM2-KO mice, though macrophage recruitment and production of H_2_O_2_ and CCL_2_ (C–C motif chemokine ligand 2) were not affected by TRPM2 [70,72].

However, we found that nerve injury-induced neuropathic pain was similarly blocked by the deletion of TRPM2 solely from DRG neurons (neuronal TRPM2) using TRPM2 conditional KO (TRPM2-CKO)) mice, in which TRPM2 in immune cells (immune TRPM2) remains intact [73]. Furthermore, there was no significant difference in macrophage and neutrophil recruitment in injured sciatic nerves between WT mice and TRPM2-CKO mice. These findings suggest that neuropathic pain was mainly carried by neuronal TRPM2 independently of immune TRPM2.

We further demonstrated the idea in antigen-induced arthritis (AIA), a rheumatoid arthritis pain model. We found that chronic arthritis pain was reduced to a similar degree between TRPM2-KO and TRPM2-CKO mice. However, inflammatory cell recruitment and cytokine production in the knee joints were largely similar between TRPM2-KO and TRPM2-CKO mice [73]. Moreover, chronic arthritis pain was rapidly reversed by pharmacologically blocking TRPM2 in the local knee joints without affecting joint inflammation. These findings support that chronic arthritis pain is mainly transduced by neuronal TRPM2 with little involvement of immune TRPM2.

We further revealed that TRPM2 was activated by PGE2 which was markedly increased in chronic arthritis pain and neuropathic pain [73]. Importantly, pain hypersensitivity evoked by PGE2 was abolished in TRPM2-KO and -CKO mice, suggesting that PGE2 induces pain hypersensitivity through activating neuronal TRPM2 [73], explaining the prominent effect of TRPM2 in chronic pain. Unexpectedly, there was no change in ADPR in the DRG and sciatic nerves, suggesting that TRPM2 is unlikely activated through ADPR or ROS signalling during chronic pain. Instead, we found that activated GoA protein, a subunit of Gαi/o protein family, caused by PGE2 directly activated TRPM2 mediating pain hypersensitivity (Figure 1). Therefore, PGE2 is a novel TRPM2 activator independent from ADPR, unlike oxidative stress that activates TRPM2 depending on ADPR.

Altogether, these new findings support that neuronal TRPM2 plays a major role in chronic pain by directly transducing pain signals acting like a pain sensor, with immune TRPM2 playing a negligible role in this process.

#### 5.1.2. Visceral Pain

TRPM2 expression was found in the afferent nerve fibres innervating the mucosa, submucosal and muscle layer across the gastric intestine (GI) tract apart from in DRG neurons. TRPM2^+^ nerve fibres were significantly increased in the colitis model induced by TNBS (2,4,6-trinitrobenzenesulfonic acid) [53,74]. TNBS also upregulated expression of cytokines and chemokines in the colon such as IL-1β, IL-6, TNFα, CXCL2, and IL-12α in wild-type mice but not in TRPM2-KO mice [53]. Visceral hypersensitivity was significantly reduced by either pharmacological inhibition or genetic deletion of TRPM2 [74], suggesting that TRPM2 is critical to visceral pain. Interestingly, TRPM2 was also expressed in the villus enterochromaffin (EC) cells in the small intestine where it acts as a sensor of oxidative stress in the mucosal environment in the GI tract [75]. Their activation released serotonin and ATP, which then excited mucosal sensory nerve fibres. This two-step activation mechanism likely mediates GI pain and nausea [75].

#### 5.1.3. Migraine

TRPM2 was also involved in migraine. In the glyceryl trinitrate (GTN) migraine mice model, GTN increased the production of ROS and cytokines (IL-1β, IL-6 and TNFα) and enhanced TRPM2 expression and function in TG neurons [76,77]. Inhibition of TRPM2 upregulation by gastrodin, a bioactive compound from the traditional Chinese medicine *Gastrodia elata* Blume (GEB), prevented migraine [76]. Furthermore, TRPM2 blockers (N-(p-amylcinnamoyl)anthranilic acid (ACA) and 2-aminoethoxydiphenyl borate (2APB)) inhibited mechanical and heat hyperalgesia in the GTN migraine mice model [77]. However, ACA and 2-APB are not specific TRPM2 blockers. The relative role of neuronal and immune TRPM2 in migraine remains to be defined.

### 5.2. TRPM2 in Seizure

Seizure is a major symptom of epilepsy. The constitutive TRPM2 channel activity was found to be protective by inhibiting seizure development and epileptogenesis [78]. Interestingly, the inhibitory effect is mediated by TRPM2 channels in microglia but not through neurons in the brain, because the deletion of microglial but not neuronal TRPM2 promoted seizure progress [78]. Surprisingly, microglial TRPM2 did not affect neuroinflammation and proinflammatory cytokines, in contrast to others’ findings showing that microglial TRPM2 promoted cytokines release mediating neuroinflammation [3,79,80,81,82,83]. Instead, it was found that microglia TRPM2 deficiency influenced synaptic structure in the hippocampus, leading to increased excitability of hippocampal neurons and synaptic transmission [78]. It remains to be determined through what factors microglial TRPM2 modulates the structure and maturation of synapses in the hippocampus.

### 5.3. TRPM2 in Ischemic Brain Damage (Stroke)

Stroke is caused by the interruption of blood supply to the brain leading to brain damage and neuronal death. Two primary mechanisms of neuronal death are excessive ROS production and Ca^2+^ overload. It was found that ROS induced neuronal death through activating ROS-sensitive TRPM2 channels [11,84,85]. Activated TRPM2 further increases Ca^2+^ load in ischemic brain cells. Furthermore, stroke causes the release of endogenous bilirubin, a byproduct of heme catabolism, from damaged blood cells [86]. Interestingly, bilirubin directly activated TRPM2 channels, leading to neuronal hyperexcitability (excitotoxicity) exacerbating Ca^2+^-dependent brain injury [86].

TRPM2 also promotes neuronal death by enhancing excitotoxicity caused by NMDAR (N-methyl-D-aspartate receptor) [87]. TRPM2 interacted with NMDAR and PKCγ. Activation of TRPM2 increased TRPM2-PKCγ association, which then further promoted membrane expression of extrasynaptic NMDAR, leading to increased NMDAR activity, excitotoxicity, and neuronal death [87,88]. Deletion of neuronal TRPM2 reduced Ca^2+^ overload, mitochondrial stress, and cell death, resulting in reduced brain injury in a mouse stroke model. Similarly, disruption of TRPM2-NMDARs or TRPM2-PKCγ interactions protected the mice against ischemic stroke [87,88].

Apart from direct contribution of neuronal TRPM2 to ischemic brain injury, TRPM2 in immune cells and microglia also promotes brain damage by inducing neuroinflammation [16].

Collectively, TRPM2 promotes ischemic brain damage though both neuronal and nonneuronal cells and direct and indirect mechanisms.

### 5.4. TRPM2 in Neurodegenerative Diseases

Neurodegenerative diseases such as Alzheimer’s diseases (AD) and Parkinson diseases (PD) are caused by progressive damage and loss of brain neurons leading to impairment in movement, memory, and other cognitive functions. Ageing is a primary risk factor. It is believed that increased oxidative stress due to declined antioxidant defence during ageing is a major mechanism of neurodegenerative diseases [89]. TRPM2 is a ROS sensor mediating oxidative stress-induced neuronal cell death and neuroinflammation [11,84]; it is thus not surprising that TRPM2 is also involved in AD and PD.

Dopaminergic (DA) neurons in the midbrain are key players in voluntary movement and emotion. DA neurons in the substantia nigra pars compacta (SNc) are more susceptible to oxidative stress, degeneration, and death than those in the ventral tegmental area (VTA) playing a prominent role in PD. Interestingly, TRPM2 was found to be preferentially expressed in the DA neurons in SNc responsible for the vulnerability of these neurons in PD. This effect was attributed to TRPM2 overactivation, leading to Ca^2+^ overload and increased ROS production in SNc DA neurons [83,90]. Deletion and inhibition of TRPM2 reduced microglia, ROS, Ca^2+^ overload, DA neuron death, and neuroinflammation and improved motor behaviour deficits [82,83,90]. Further in vitro experiments in cultured cells suggest that neuronal and microglial TRPM2 are involved in neuronal cell death, microglia activation, and neuroinflammation, respectively, by selectively knocking down TRPM2 in microglia and differentiated DA neurons [82]. More in vivo experiments are required to verify the finding through spatial deletion of neuronal and microglial TRPM2, respectively.

AD is caused by abnormal accumulation of amyloid β (Aβ) plaques and tau tangles leading to neuronal dysfunction and death. Oxidative stress and neuroinflammation in the hippocampus contribute to the onset and progression of AD. Aβ peptide caused oxidative neurotoxicity and apoptosis in the hippocampal and striatal neurons and the effect was prevented in TRPM2-KO mice or by downregulating TRPM2 function [8,91]. Aβ-42 also activated microglial cells and induced TNFα generation depending on TRPM2 [92,93]. Aβ-42 caused TRPM2 activation through generated ROS and activation of PARP-1 [93]. In support of this mechanism, antioxidant treatment inhibited TRPM2 activation and reduced neuronal death and microglial activation [94]. Memory deficits in the AD mouse model were also reversed in TRPM2-KO mice [92]. Therefore, ROS generated by Aβ peptide activates TRPM2, which then induces neuronal death, neuroinflammation, and oxidative stress forming positive feedback.

Taken together, TRPM2 plays a key role in neuronal death and neuroinflammation in AD and PD through the cooperative actions of neuronal and microglial TRPM2. However, the respective role of neuronal and immune TRPM2 in the progression of these neurodegenerative diseases remains to be clarified in the future.

It is noteworthy that many other factors, especially Ca^2+^ permeable ion channels, are also involved in these neurological diseases apart from TRPM2. For example, considerable evidence supports that chronic pain is also mediated by TRPV1 [95], TRPA1 (transient receptor potential ankyrin 1) [95,96], TRPM3 [95,97,98], TRPC3 [99,100] and TRPC5 [101] channels. A recent research demonstrates that ischaemic neurotoxicity in stroke also involves potentiation of ASIC1a (acid-sensing ion channel 1a) channels by glutamate in addition to the NMDA receptor [102]. Ca^2+^ signalling initiated by these Ca^2+^-permeable channels orchestrates multiple interlocking downstream events such as neurotoxicity, ROS generation, cytokines/chemokine and neuropeptide release, neuroinflammation, and gene dysregulation. These, and other factors, act together with TRPM2 to promote disease development and progression.

## 6. Concluding Remarks

TRPM2 is highly expressed in the neuroimmune system contributing to neuronal function and neuronal death through neuronal TRPM2 and mediating immune-inflammatory response and neuroinflammation through immune TRPM2. Activation of TRPM2 triggers simultaneous activation of both the nervous and immune systems which then crosstalk with each other, boosting mutual functions (Figure 1). This feed-forward positive activation model underlies the broad role of TRPM2 in many pathophysiological conditions such as infection and inflammation, thermal sensing and regulation, pain, stroke, and neurodegenerative diseases. This model also likely determines many other disease conditions such as cancer [103], atherosclerosis [104], myocardial infarction [105], ischemic liver, and kidney injury [50,106,107]. More research is required to further understand the respective role of neuronal and immune TRPM2 in these diseases and the molecules activated by TRPM2 responsible for neuroimmune crosstalk. Apart from the positive feedback regulation mediated TRPM2, TRPM2 also induces negative feedback regulation, exerting protective effect depending on different tissue contexts and disease models. Research is also required to understand the molecular determinants underlying the bidirectional effect of TRPM2. TRPM2 is not only expressed in the neuroimmune system, but also in many other tissue and organs. The complex interaction of the neuroimmune system with other TRPM2-positive cells and tissues may underlie the complex bidirectional effect of TRPM2. Further understanding of these questions will provide the rationale for better targeting TRPM2 for the treatment of the diseases mediated by TRPM2.

## Data Availability

This review did not report any data and, therefore, the statement of data availability is not applicable.
